# Methylcholanthrene-Induced Sarcomas Develop Independently from NOX2-Derived ROS

**DOI:** 10.1371/journal.pone.0129786

**Published:** 2015-06-15

**Authors:** Maarten A. Ligtenberg, Özcan Çınar, Rikard Holmdahl, Dimitrios Mougiakakos, Rolf Kiessling

**Affiliations:** 1 Department of Oncology and Pathology, Cancer Center Karolinska, Karolinska Institutet, Stockholm, Sweden; 2 Division of Medical Inflammation Research, Department of Medical Biochemistry and Biophysics, Karolinska Institutet, Stockholm, Sweden; 3 Department of Internal Medicine 5, Haematology and Oncology, University of Erlangen-Nuremberg, Nuremberg, Germany; Ohio State University, UNITED STATES

## Abstract

Reactive oxygen species (ROS) produced by the inducible NADPH oxidase type 2 (NOX2) complex are essential for clearing certain infectious organisms but may also have a role in regulating inflammation and immune response. For example, ROS is involved in myeloid derived suppressor cell (MDSC)- and regulatory T cell (T_reg_) mediated T- and NK-cell suppression. However, abundant ROS produced within the tumor microenvironment, or by the tumor itself may also yield oxidative stress, which can blunt anti-tumor immune responses as well as eventually leading to tumor toxicity. In this study we aimed to decipher the role of NOX2-derived ROS in a chemically (by methylcholanthrene (MCA)) induced sarcoma model. Superoxide production by NOX2 requires the p47^phox^ (NCF1) subunit to organize the formation of the NOX2 complex on the cell membrane. Homozygous mutant mice (NCF1*^/^*) have a functional loss of their super oxide burst while heterozygous mice (NCF1*^/+^) retain this key function. Mice harboring either a homo- or a heterozygous mutation were injected intramuscularly with MCA to induce sarcoma formation. We found that NOX2 functionality does not determine tumor incidence in the tested MCA model. Comprehensive immune monitoring in tumor bearing mice showed that infiltrating immune cells experienced an increase in their oxidative state regardless of the NOX2 functionality. While MCA-induced sarcomas where characterized by a T_reg_ and MDSC accumulation, no significant differences could be found between NCF1*^/^* and NCF1*^/+^ mice. Furthermore, infiltrating T cells showed an increase in effector-memory cell phenotype markers in both NCF1*^/^* and NCF1*^/+^ mice. Tumors established from both NCF1*^/^* and NCF1*^/+^ mice were tested for their *in vitro* proliferative capacity as well as their resistance to cisplatin and radiation therapy, with no differences being recorded. Overall our findings indicate that NOX2 activity does not play a key role in tumor development or immune cell infiltration in the chemically induced MCA sarcoma model.

## Introduction

Novel immunotherapeutic strategies are largely tested in transplantable murine tumor models. However, preclinical success is often difficult to translate into clinical efficacy especially when applied to cancer patients with slowly progressing malignancies [1]. These apparently contradictory observations between transplantable tumor models and cancer patients could largely be explained by the ability of slowly progressing tumors to efficiently shape immune responses resulting in diminished anti-tumor reactivity [2].

Primary carcinogen-induced murine tumors better resemble disease dynamics of slowly progressing human tumors. The model of chemically induced sarcomas is the prototype for a strongly immunogenic tumor and was used in landmark experiments to define tumor transplantation antigens [3–5]. More recently, studies of carcinogen-induced tumors carried out in various knockout mice validated the immunoediting hypothesis [6]. This model has been particularly useful in demonstrating the role of both adaptive and innate immune cells in eliminating tumor cells. Immunodeficient mice challenged with methylcholanthrene (MCA) develop sarcomas more frequently and more rapidly as compared to their immunocompetent counterparts [7].

Tumor induced immunosuppression, as manifested in cancer patients and mice with large tumors, is known to be co-mediated by lymphoid and myeloid cells. T regulatory cells (T_regs_) and so-called myeloid derived suppressor cells (MDSC) are often increased in number and immunosuppressive capacity in both patients and pre-clinical tumor models. They employ a broad arsenal of mechanisms to blunt T and NK cell responses. These mechanisms include the secretion of suppressive cytokines, the depletion of metabolites critical for T cell functions, and the production of reactive oxygen species (ROS) [8,9].

Oxidative stress is a common phenomenon in malignancies [10] and represents a major contribution to tumor induced immunosuppression [11]. Abundant ROS production responsible for oxidative stress has three main sources. External factors, such as UV radiation, generate free radicals that have been linked to skin carcinogenesis [12]. Next, mitochondrial dysfunction also promotes ROS production [13]. Finally, increased NADPH oxidase (NOX) activity can lead to tumor-associated oxidative stress [14]. ROS can drive tumorigenesis though the activation of oncogenes such as c-myc and NF-kB, through up-regulation of pro-EMT proteins such as matrix metalloproteinases, and through direct DNA damage producing somatic mutations that further potentiate tumorigenesis [15]. NOX2 is responsible for the inducible oxidative burst, which is utilized by phagocytic cells for the inactivation of bacterial and fungal infections [16]. However, increased NOX2 expression has been described in several malignant entities, including breast- colon- and prostate cancer and leukemia [17–20].

In addition to their direct effects on the cancer cells’ biology, ROS exhibit indirect effects by suppressing intrinsic anti-tumor immune responses [11]. NOX2 is expressed in MDSC and T_regs_, and is used by these immune subsets to exert their suppressive function. Exposure of T and NK cells to oxidative stress leads to CD3-ζ chain loss, an impaired nuclear NFκB translocation and increased cell death [11]. Additionally, ROS seem to lend a survival advantage to T_regs_, leading to an increased suppressive feedback loop [21].

Here, we sought to determine whether ROS production by NOX2 has any impact on the malignant potential and/or the anti-tumor immune responses in chemically-induced sarcomas. To this end, we have taken advantage of a mouse model in which NCF1, a gene encoding the subunit p47^phox^ of the NOX2 complex, has a single nucleotide mutation leading to a loss of function [22]. We report that MCA-induced tumors developed at a similar rate in litter-paired homozygous and heterozygous NCF1 mutant mice. Furthermore, we observed a sarcoma-induced accumulation of MDSC and T_regs_ irrespective of the NCF1 mutational status. For elucidating the potential effects of the (autocrine) NOX2 activity on the malignant features we established cell lines from primary tumors. Those were characterized in terms of growth, invasiveness, and radio-chemoresistance without detecting significant differences. Overall, our studies indicate that NOX2-derived ROS neither play a substantial direct nor an indirect role in the chemically-induced MCA tumor model.

## Material and Methods

### Mice and MCA-induced tumor model

NCF1m1J mutant mice (NCF1*) NCF1* mice were previously backcrossed into C57Bl/10.Q/rhd mice. They were genotyped as previously described and crossed to generate heterozygous NCF1*^/+^ and homozygous NCF1*^/^* mutant mice [22]. To induce sarcoma tumorigenesis, mice (approximately 20 g, 8–16 weeks old) were treated by intramuscularly injection of 0.025 ml of an emulsion consisting of 3-methylcholanthrene (Sigma-Aldrich)-Corn oil (5mg/ml) in the right hind leg. Mice were observed weekly for the development of intramuscular tumor development from 70–160 days. Tumors were harvested and mice sacrificed as described below. From harvested tumors, cell lines were established using typical mechanical dissociation and Collagenase IV/DNAase/Trypsine treatment followed by single cell suspension generation by passage through filter prior to seeding into T-25 flasks with complete RPMI medium containing 10% FBS 10mM nonessential amino acids, 50 units/ml penicillin, 50 μg/ml streptomycin.

### Ethics statement

Animals were maintained at the animal facility at the department of Microbiology, Tumor and Cell Biology according to the guidelines of the Regional Animal Ethics Committee (Stockholms Norra Djurförsoksetiska Nämnd Avdelning 2) which specifically approved this study (permit number: N283/09). Conventional animal housing was used with 12-hours dark/light cycles and eight mice were kept per plastic cage with wood-chip bedding. Pelleted food and water were provided *ad libitum*. Animal suffering was minimized by reducing the required injections to one i.m. delivery of MCA-Corn oil and by further strict adherence to the guidelines as well as daily animal health monitoring. During the study no unexpected deaths were observed. No anaesthetics or analgesics were used during this study. Animals were euthanatized using exposure to 100% carbon dioxide in a sealed chamber when ethical experimental endpoints were met. The endpoint was determined through the use of caliper measurement of the tumor diameter that was set at a maximum size of 1 cm, as approved by the local animal ethics committee.

### Antibodies and Flow Cytometry

The following monoclonal anti-mouse antibodies were used: CD4 (clone GK1.5), CD8 (clone 53–6.7), CD3 (clone 17A2), CD44 (clone IM7), CD69 (clone H1.2F3), CD62L (clone MEL-14), Ly6C (clone HK1.4), Ly6G (clone 1A8), CD11b (M1/70), IL-17a (clone TC11-18H10.1), IL-4 (clone 11B11), IFN-γ (clone XMG1.2), FoxP3 (MF-14), CD25 (clone PC61), Gr1 (clone RB6-8C5), CD45 (clone 30-F11) (BioLedgend, San Diego, CA). Single cell suspensions we stained prior to flow cytometry-based acquisition. Cells were additionally stained with fixable aqua dead cell stain kit (Life Technologies, Carlsbad, CA). For staining spleens and tumors were submitted to mechanical dissociation by passing through a 70 μm filter. The tumors were additionally treated as described above to generate a single cell suspension. All samples were treated with red blood cell lysis buffer (BioLegend) for 5 minutes and washed twice prior to further staining. For profiling T cell cytokines, single cell suspensions from blood, spleen, and tumor were stimulated with 2.5 μg/ml of phytohaemagluttinin (PHA, Sigma-Aldrich, St. Louis, MO) for one hour prior to addition of Golgi plug (BD Bioscience, San Jose, CA) for an additional stimulation of 3 hours at 37°C. Cells were stained with cell surface markers and intracellular cytokine staining was done in accordance with the manufacturer’s instructions using the Cytofix/Cytoperm and Permwash Kit (BD Bioscience). Cells were acquired on a LSRII cytometer (BD Bioscience) and the data were analyzed using FlowJo v9 (Tree Star Inc. Ashland, OR).

### Reactive Oxygen Species detection

Oxidative burst was evaluated by labeling splenocytes in single cell suspension with 3μM dihydro-rhodamine 123 (DHR123; Life Technologies) at 37°C for 10 minutes in PBS (Life Technology). Cells were activated by stimulation with 200 ng/ml phorbol 12-mystate 13-acetate (PMA, Sigma-Aldrich) for 20 minutes prior to acquisition.

To measure oxidative state in blood, spleen and tumor, cells were stained with antibodies as described above followed by incubation for 20 additional minutes with 3 μM DHR123 (Life Technologies) in PBS at 37°C followed by immediate acquisition using LSRII cytometry.

### Proliferation assay and chemotherapy resistance

MCA tumor cell lines were seeded into E-Plate 16 (ACEA Biosciences, San Diego, CA) once they had reached log phase growth in T-75 cell culture flask. 200 μl of complete RPMI medium (RPMI GlutaMAX, 10% FCS and 1% PenStrep) containing 5x10^4^ cells/ml of cells were transferred to E-Plates. E-Plates were incubated for 30 minutes to allow for settling of cells and then transferred into the xCelligence RTCA DP Analyzer (ACEA Bioscience) and cell indexes were recorded every 30 minutes over a 7-day period. Growth rate (slope) was determined using linear regression analysis where 95% R^2^ has been observed between two time points at the logarithmic growth phase of the cells. Cisplatin (5 μg/ml) was added when cells were determined to enter the log-phase growth. Death rate induced by Cisplatin was determined similarly to the growth rate from the moment effect of cisplatin was observed.

### Invasion assay

MCA tumor cells were kept on starvation medium (RPMI GlutaMAX with 1% PenStrep) for 6 hours prior to seeding 1x10^4^ cells/well into CIM-Plate 16 (ACEA Biosciences). The upper chamber of CIM-Plate 16 was coated with 40 μl of 2.5% Matrigel basement membrane matrix (BD Bioscience) diluted in ice cold PBS followed by incubation for 4 hours for matrigel polymerization. 175 μl of complete medium (RPMI GlutaMAX, 10% FCS and 1% PenStrep) was added to the lower chamber of CIM-Plate 16 prior to assembly with the upper chamber to which an additional 30 μl of starvation medium was added in the upper chamber. CIM-Plate 16 were placed in the xCelligence RTCA DP Analyzer and incubated for 3 hours to allow the CIM-Plate 16 membrane surface to reach equilibrium with media. Once tumor cells were seeded into the plate in four replicates cell indexes were recorded every 15 minutes for up to 180 hours. Invasiveness was evaluated based on the initiation of increasing cell index beyond 0.005. From this moment till slope was determined as invasion rate.

### Radiation resistance and cellular proliferation (XTT)

MCA tumor cell lines were seeded into 96-well plates with the concentration of 100 cells/well as five replicates for each cell line, for each day, as control and treatment plates. Plates were incubated 2 hours at 37°C. Subsequently, the baseline metabolic was determined using Cell Proliferation Kit II (XTT) (Roche, REF—11 465 015 001) according to manufacturer’s protocol. Meanwhile treatment plates were treated with 2 gray gamma radiation using a Cesium-137 source (Instrument AB Scanditronix; Husbyborg, Uppsala, Sweden) and returned to the 37°C with 5% CO_2_. XTT measurement was repeated on days 3, 6 and 9. Relative survival rates for individual cell lines were calculated by dividing XTT intensity of treated wells to the non-treated controls.

### Statistics

Analysis of data was performed using Prism GraphPad 5. For a comparison of differences between two nonparametric data sets Mann-Whitney test was used. Survival of mice post MCA challenge was compared using Log-rank (Mantel-Cox) test and displayed as Kaplan-Meier curves. P values under 0.05 were considered as statistically significant.

## Results

### NCF1 mutational status has no overall impact on MCA-induced sarcoma growth

**Fig 1:**
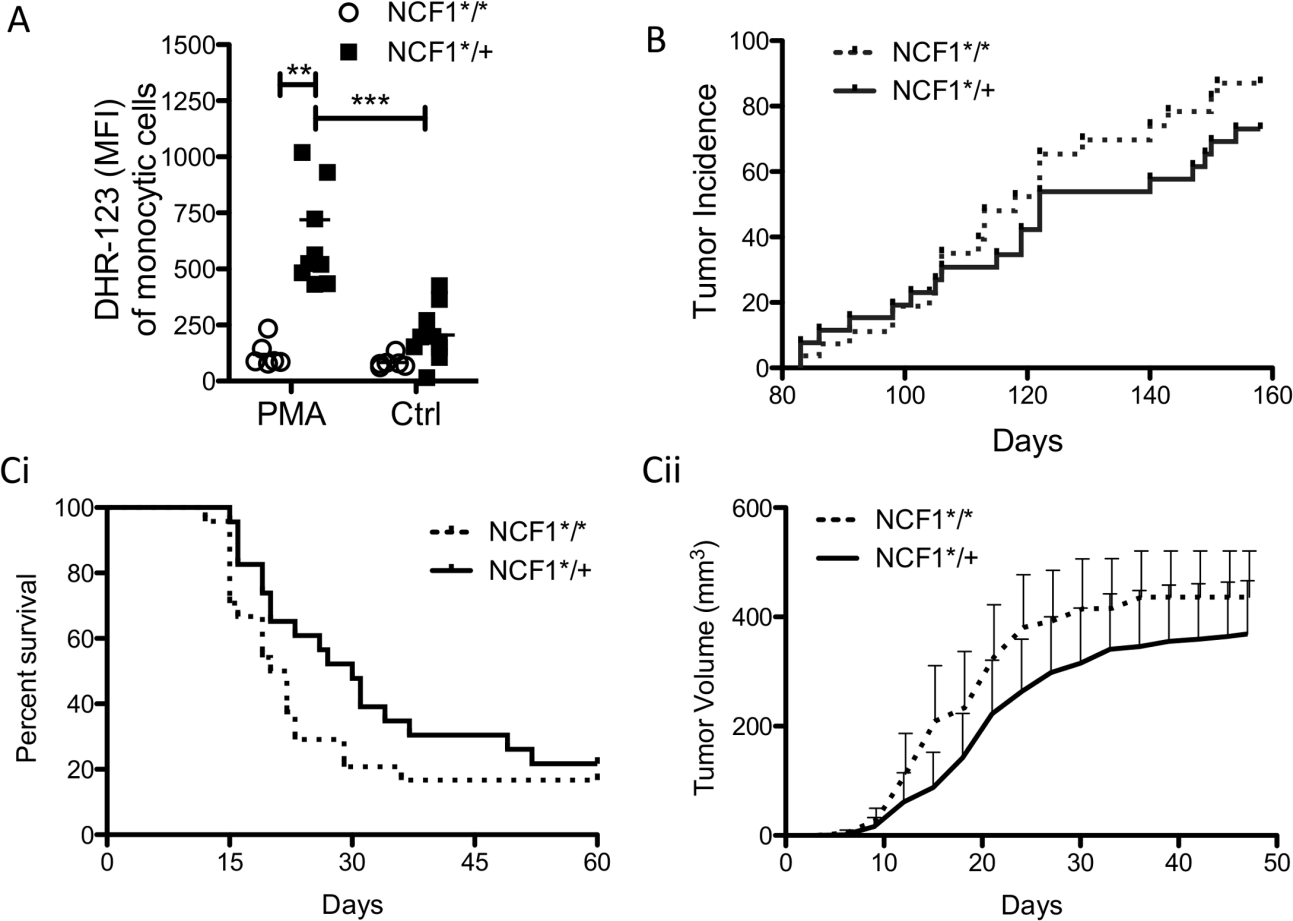
Growth of MCA induced tumors is not affected by NCF1 mutation. (A) NCF1*^/^* (n = 6) and NCF1*^/+^ (n = 10) mice were tested for their ability to generate superoxide through stimulation of NOX2 with PMA. (B) NCF1*^/^* and NCF1*^/+^ mice (n = 54, 27 per group) were injected with 125 μg MCA dissolved in 25 μl of corn oil. Tumor incidence was followed through palpation of the injected muscle site. (C) Tumors were resected from MCA injected NCF1*^/^* (n = 6) and NCF1*^/+^ (n = 6) mice and passaged once in WT C57Bl/6 mice prior to transplantation of 5^3^ mm tumor into four WT C57Bl/6 (n = 48) mice in which (Ci) survival (Cii) and tumor growth were monitored.

Mice with a single point mutation in the NCF1 gene, which encodes for the protein p47^phox^, were backcrossed onto C57Bl/10.Q background. Mice homozygous for the NCF1 mutation (NCF1*^/^*) have non-functional p47^phox^ and are incapable of assembling NOX2, due to deficient interaction with the p22 protein, to generate super oxide [23]. To confirm this functional deficit, splenocytes from homozygous NCF1*/* and heterozygous (NCF1*^/+^) mutant mice were labeled with DHR-123 and treated with NOX2 stimulating PMA. Gating on monocytic cells (S1 Fig) indicates that the NCF1*^/^* mice are incapable of responding with ROS production upon stimulation in contrast to NCF1*^/+^ mice (Fig 1A).

The ability of macrophages to generate superoxide through NOX activation has been shown to play an important role in resolving inflammatory severity in arthritis models [22], which may also apply to inhibiting anti-tumor responses in tumorigenesis. To examine the impact of non-functional NOX2 in a chemically-induced tumor model NCF1*^/^* (n = 27) and NCF1*^/+^ (n = 27) mice were injected intramuscularly with MCA. Tumor incidence was followed over a period of 160 days (Fig 1B) with no significant differences being found.

Next, we hypothesized that immunoselection during tumor development may have influenced the immunogenicity of the MCA-induced tumors differently in mice with functional as compared to non-functional NOX2. This was tested by transplanting six tumors generated from either in NCF1*^/^* or NCF1*^/+^ mice into four wild type C57Bl/6 mice. However, no difference as observed between the tumors derived from mice with functional as compared to non-functional NOX in terms of survival and tumor growth (Fig 1C).

### MCA tumors promote recruitment and generation of suppressive immune cells

**Fig 2:**
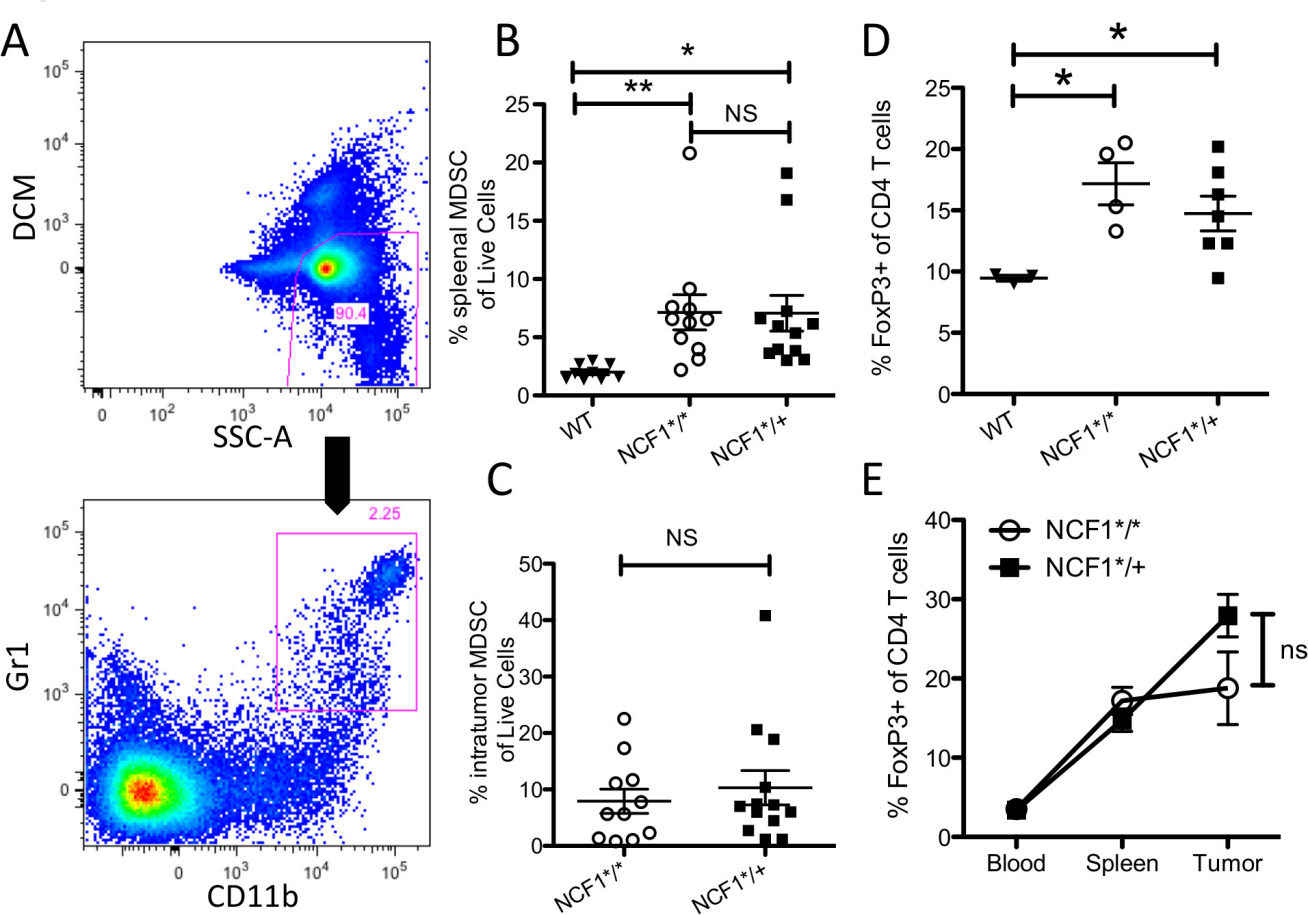
MCA induced tumors accumulate immune regulatory cells. **(**A) MDSCs as characterized by CD11b+ and GR1+ were gated from splenocytes or tumor samples as a percentage of live cells. (B) Percentage of live splenocytes that are MDSCs in tumor bearing NCF1*^/^* and NCF1*^/+^ mice compared to WT mice. (C) Percentage of live cells which are MDSC in tumors from both NCF1*^/^* and NCF1*^/+^ mice. (D) Percent FoxP3+ cells of CD4+ cells in spleens of tumor bearing mice compared to WT mice. (E) Percent T_reg_ cells in the blood, spleen and tumor of MCA tumor bearing mice.

Tumorigenesis is associated with chronic inflammation and immunosuppression. We were able to confirm that MCA induced tumor lead to accumulation of MDSCs in the spleen (CD11b+, Gr1+) (Fig 2A). MCA-induced tumors significantly increased the amount of MDSCs present in the spleen in both NCF1*^/^* and NCF1*^/+^ mice compared to tumor free wild type mice (Fig 2B). No differences were found in the frequency of intratumoral MDSCs between NCF1*^/^* and NCF1*^/+^ mice (Fig 2C). Tumorigenesis is also known to be paralleled by an increase of T_reg_. In fact, sarcomas induced by MCA increase the frequency of T_reg_ (FoxP3+ of CD3+, CD4+ cells, Sup. Fig 2A) in the splenocytes significantly in both NCF1*^/^* and NCF1*^/+^ mice as compared to WT mice (Fig 2D). Interestingly, frequency of T_reg_ increased significantly from peripheral blood to the spleen and from the blood to the tumor in both NCF1*^/^* and NCF1*^/+^ indicating the accumulation of T_reg_, (S2 Fig Bi, Bii) though no difference was found between NCF1*^/^* and NCF1*^/+^ mice in the tumor (Fig 2E).

### T cell infiltration pattern in MCA-induced tumors is not affected by the NCF1 mutational status

**Fig 3:**
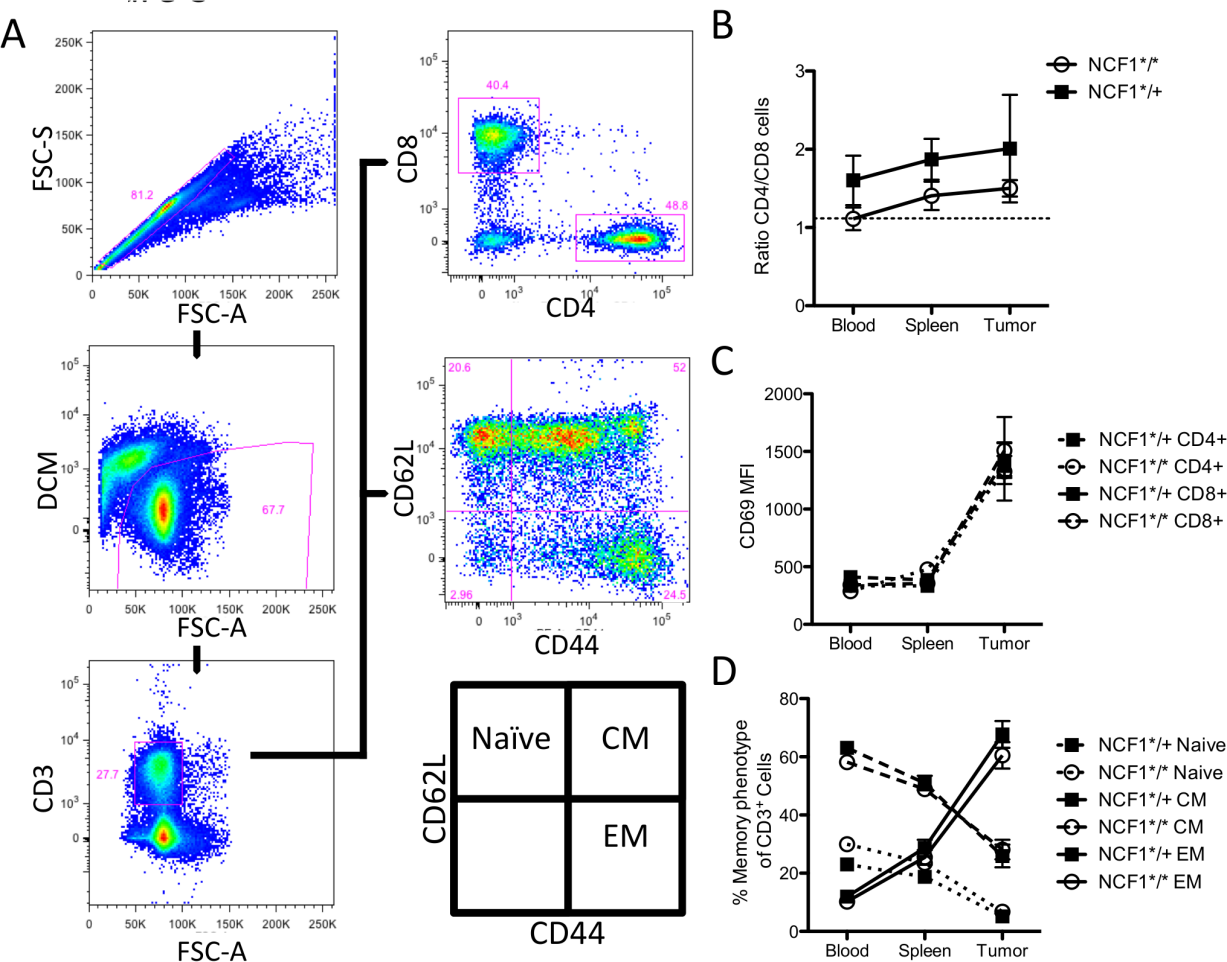
T cells are not affected differently in NCF1*^/^* compared to NCF1*^/+^ tumor bearing mice with regard to their CD4/CD8 ratio, activation status or memory phenotype. (A) T cells were gated as singlet cells that were not stained for dead cell marker but positive for CD3. (B) The ratio of CD4+ to CD8+ was gated in blood, spleen and tumor in NCF1*^/^* and NCF1*^/+^ mice, with the dotted line representing the ratio found in WT C57Bl/6 spleenocytes. (C) Activation marker CD69 was stained on blood, spleen and tumor resident T cells. (D) Blood, spleen and tumor resident T cells were stained for CD62L as well as CD44 to indicate their memory phenotype.

Next we examined the phenotype of the tumor infiltrating T cells in the MCA induced NCF1*^/^* and NCF1*^/+^ mice. T cells were gated for memory markers (CD62L and CD44) and CD4/CD8 ratio by gating on live singlet CD3+ T cells (Fig 3A).The CD4/CD8 T cell ratio was compared between tumor bearing NCF1*^/^* and NCF1*^/+^ mice (Fig 3B). T cells become activated when they enter the tumor microenvironment (Fig 3C); this is significantly the case in both mutant and heterozygous NCF1 mice though no difference in activation was found between the NCF1*^/^* and NCF1*^/+^ in the tumor (S3A Fig). The memory phenotype of T cells follows an expected pattern of a significant increase in effector memory T cells from the blood to the spleen and highest within the tumor, but with no differences between NCF1*^/^* and NCF1*^/+^ mice (S3B Fig). While these cell subsets increase in frequency, central memory and naïve cells decreased (Fig 3D).

**Fig 4:**
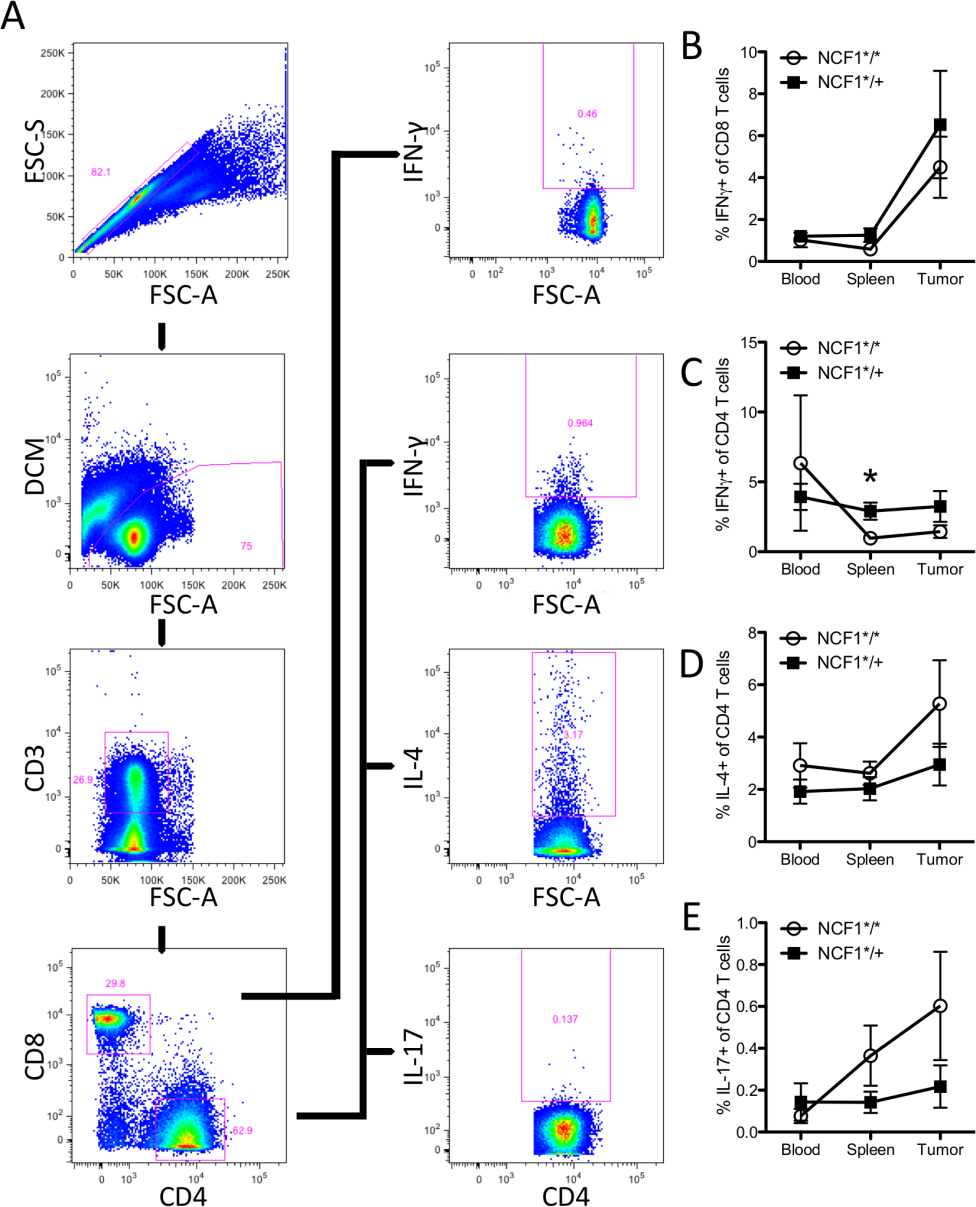
No difference was found between tumor bearing NCF1*^/^* and NCF1*^/+^ mice in T cell cytokine production. (A) Single cell suspensions of blood, spleen and tumor samples were stimulated with PMA for one hour followed by three hours with GolgiPlug prior to intracellular staining. T cells were gated as follows; singlet to live to CD3 positive and the split on CD4 or CD8 positive cells. (B) Intracellular IFN- γ response in CD8+ T cells. (C) Intracellular IFN- γ response in CD4+ T cells. Mann-Whitney statistical test; * P < 0.05. (D) Intracellular IL-4 response in CD4+ T cells. (E) Intracellular IL-17 response in CD4+ T cells (E).

While the fraction of T cells may not have decreased in NCF1*^/+^ mice as compared to NCF1*^/^* mice their functionality may have been effected due to mutational status. This was tested by a short-term stimulation of T cells with PMA followed by intracellular staining of CD8+cells for IFN-γ and of CD4+ cells for IFN-γ, IL-4, and IL-17 (Fig 4A). The proportion of IFN-γ+ cells in CD8+ T cells did not differ between NCF1*^/^* and NCF1*^/+^ mice, but showed a marked (adequate) increase within the tumor environment (Fig 4B) that matched the kinetics of the CD69 activation marker expression (Fig 3C). The secretion of particular cytokines such as IFN-γ, IL-4 or IL-17 can define T helper subsets: Th1, Th2 and Th17 respectively. We examined those subsets in NCF1*^/^* and NCF1*^/+^ mice bearing MCA-induced tumors. The fraction of CD4 T cells secreting IFN-γ was found to be significantly higher (p<0.05) in the spleens of NCF1*^/+^ mice and showed a similar trend at the tumor site (Fig 4C). When comparing the ratio of Th1 to Th2 cells there was a trend in the NCF1*^/^* mice to have a lower ratio than NCF1*^/+^ mice which was significant in the spleen (p<0.05) (S4 Fig). NCF1*^/^* mice have a trend towards an increased Th2 response in all compartments (Fig 4D). Only a small fraction of CD4 T cells secreted IL-17 with NCF1*^/^* mutant mice trending towards more IL-17 positive CD4 cells (Fig 4E).

### NCF1 mutational status does not influence oxidative state in the tumor

**Fig 5:**
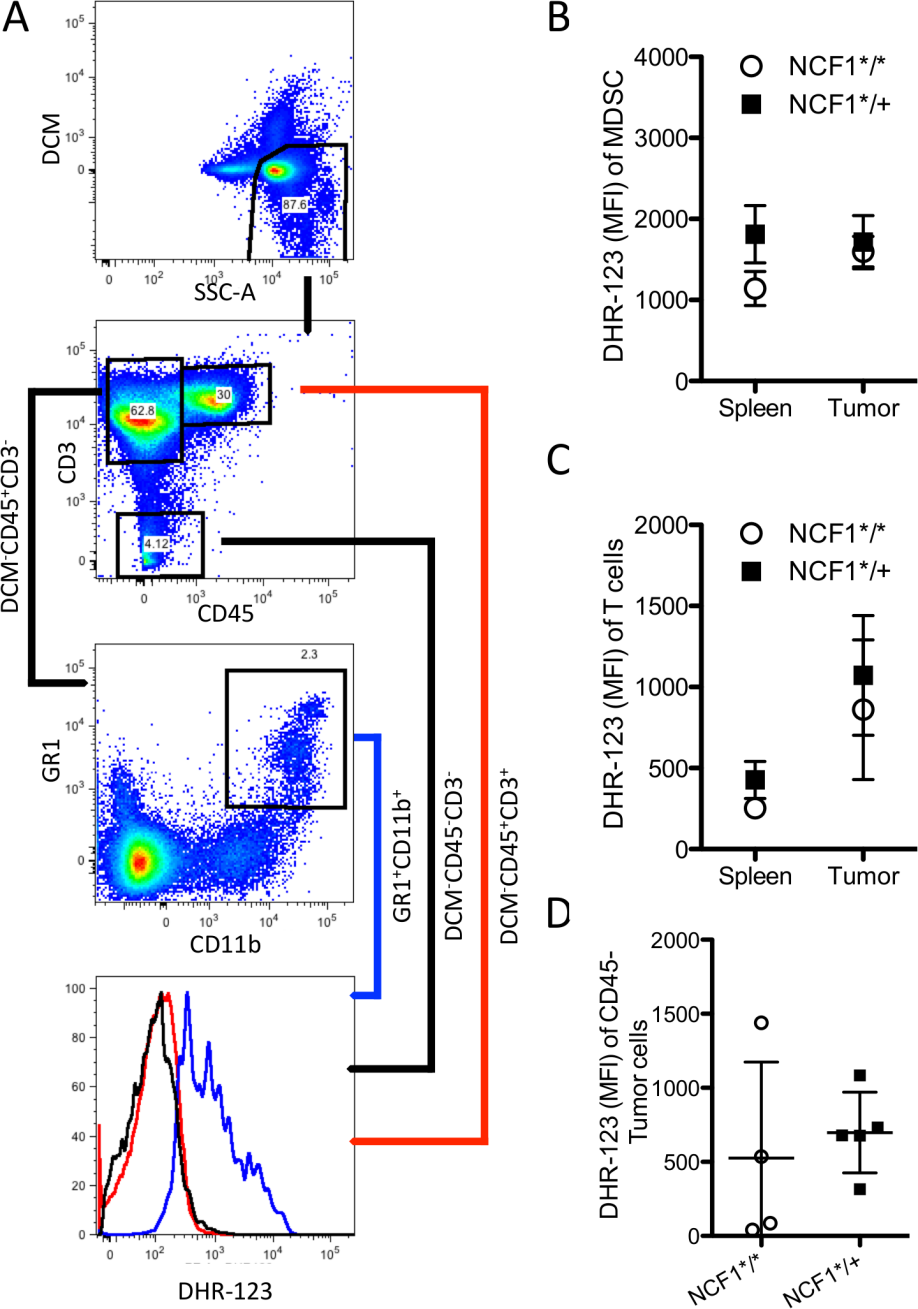
Oxidative state does not differ in immune cell subsets or tumors from NCF1*^/^* and NCF1*^/+^ tumor bearing mice. (A) Cells were gated on live followed by CD45 positive both CD3 negative followed by CD11b positive and GR1 positive for MDSC, CD45 positive CD3 positive indicated T cells and CD45 negative live cells were considered tumor cells. (B) DHR-123 MFI of MDSCs as well (C) T cells and (D) tumor cells were determined in NCF1*^/^* (n = 4) and NCF1*^/+^ (n = 5) tumor bearing mice.

NOX2, when assembled, produces superoxide that represents a mode of immunosuppression exerted by MDSCs on T cells [24]. To study whether cells in NCF1*^/^* mice experience less oxidative stress than cells in NCF1*^/+^ mice, spleen and tumor samples were stained with DHR-123 as well as antibodies for T cells (CD45+CD3+), MDSCs (CD45+CD3-CD11b+GR1+), and tumor cells (DCM-CD45-) (Fig 5A). MDSCs in the spleen and in the tumor exhibit similar intracellular ROS levels (Fig 5B). Tumor infiltrating T cells tend to experience increased oxidative stress (Fig 5C), while no differences could be found between NCF1*^/^* and NCF1*^/+^ MCA-induced CD45- tumor cells (Fig 5D).

### In vitro characterization of MCA induced tumors generated in NCF1*^/^* and NCF1*^/+^ mice

**Fig 6:**
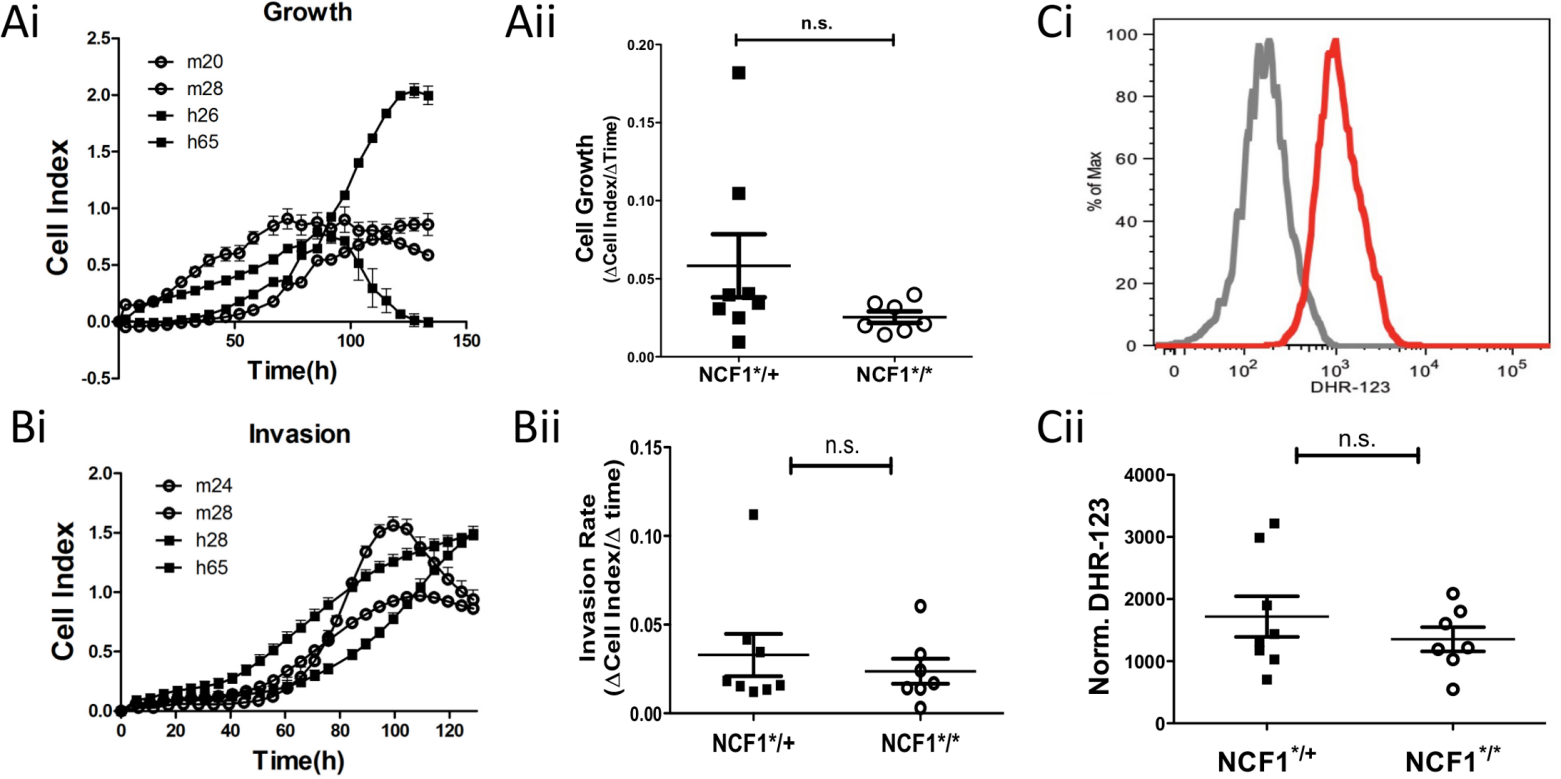
In vitro characteristics of NCF1*^/^* and NCF1*^/+^ MCA induced tumors. Tumors cell lines were generated from MCA induced tumors by mechanical dissociation followed by treatment with Collagenase IV and Trypsin. Tumor cells were plated at low confluence on Xcelleigence E-plates and (Ai) growth was monitored over time and (Aii) cell growth rate was determined based on the logarithmic growth phase. (Bi) Tumor cells were seeded onto CIM-16 xCELLigence plates with serum medium gradient separated by matrigel plug to evaluate invasion for which the (Bii) invasion rate was compared between NCF1*^/^* and NCF1*^/+^ cell lines. (C) Tumor cells were labeled with DHR-123 and analyzed by flow cytometry.

Oxidative stress in tumorigenesis has been shown to have an impact on the proliferative as well as invasive properties of tumor cells. Fifteen in vitro tumor cell lines were generated from tumors extracted from MCA inoculated NCF1*^/^* and NCF1*^/+^ mice. Their capacity to proliferate was found to be independent of the NCF1 mutational status (Fig 6A). To assess whether these tumor cell lines had different invasive characteristics they were seeded onto CIM-16 xCELLigence plates and invasiveness was determined as detailed in the material and methods section (Fig 6B). No differences could be found between tumors generated in the NCF1*^/^* as compared to NCF1*^/+^ mice. While many cancers display oxidative stress as a result of mitochondrial dysfunctions some types of prostate carcinomas [19] and melanoma [25] have been found to over-express NOX2, leading us to evaluate whether the tumor cell lines produced amounts of oxidative stress related to their NCF1 mutation status. Tumor cell lines were labeled with DHR-123 followed by acquisition by flow cytometry. While we could confirm that the tumors were able to oxidize the dye, this was independent of NCF1*^/^* or NCF1*^/+^ mutational status (Fig 6C). This lead us to conclude that the MCA induced sarcoma cell lines did not depend on NOX2 for oxidative stress generation.

**Fig 7:**
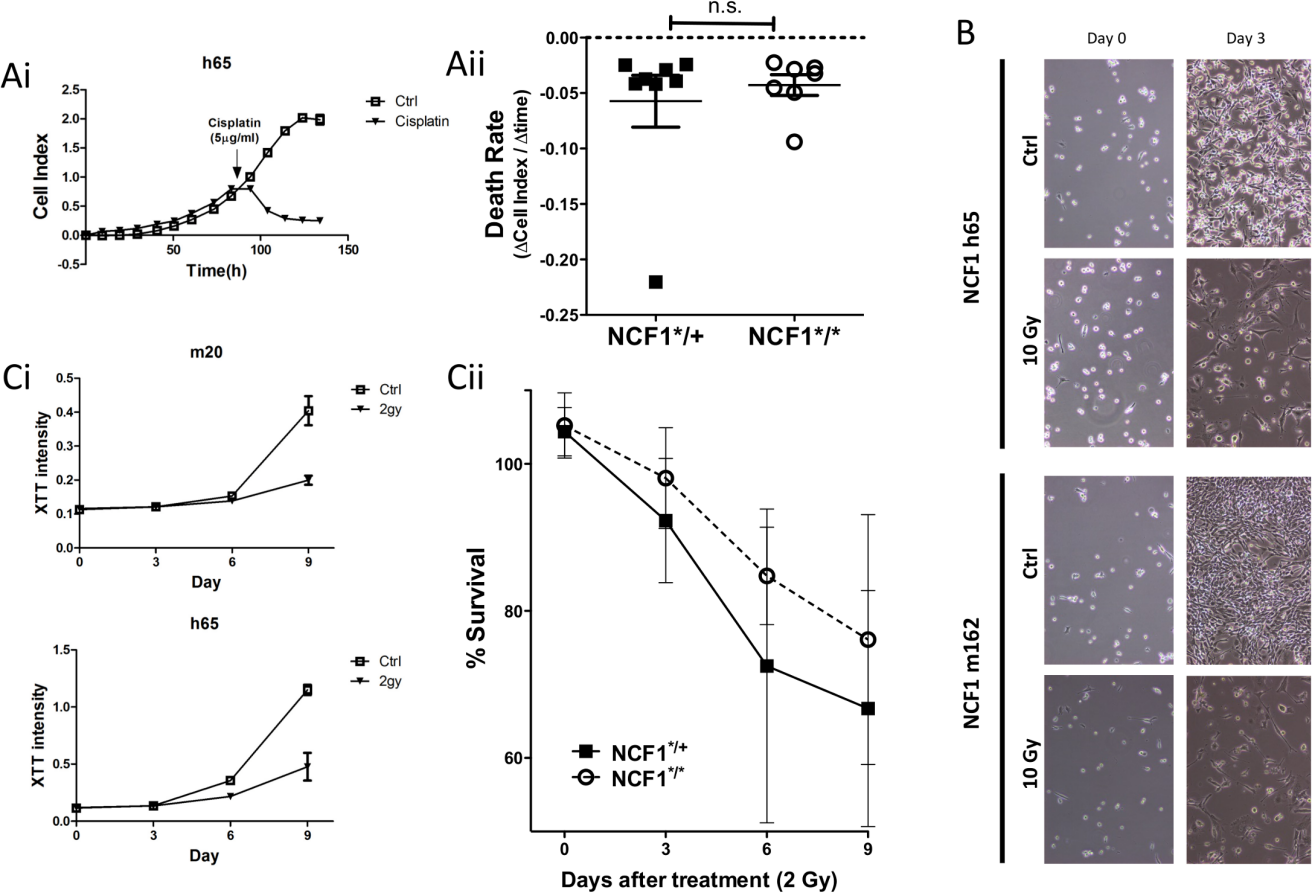
Non-functional NOX2 does not change susceptibility to cisplatin or radiotherapy. (Ai) Cells were seeded in E-plates and treated with 5μg/ml of cisplatin at log phase growth. (Aii) Rate of cell death after addition of cisplatin was compared between NCF1 tumor cell lines, derived from MCA induced tumor in NCF1*^/^* and NCF1*^/+^ mice. (B) NCF1 tumor cell lines were treated with 10 gray and morphology of the cells was followed for three days. (Ci) 100 tumor cells were seeded in plates and treated with 2 gray and followed over 6 or 9 days. Metabolic activity was measured with XTT as an indication of survival with treatment over control (Cii) indicating survival.

Chemotherapy or radiotherapy rely amongst others on oxidative stress to kill tumor cells. Tumors exposed previously to chronic (mild) oxidative stress become more resilient towards chemo- and/or radiotherapy due to intrinsic compensatory mechanisms. To evaluate whether tumors induced in NCF1*^/^* mice have alterations regarding their chemoresistance, we compared the rate of cell death after treatment with clinically relevant concentrations of cisplatin (5 μg/ml) (Fig 7A). In addition, NCF1 tumor cell lines were treated with γ radiation at 10 Gy (Fig 7B) which led to mitotic cell death. Next, we applied a single 2 Gy dose, and followed the metabolic activity up to nine days in both homozygous and heterozygous mutant tumor cell lines. Survival was calculated based on the percentage of survival over 6 days (Fig 7C). Overall, this data indicates that lack of NOX2 activity had no impact on the radio-chemo-sensitivity of MCA-induced sarcomas.

## Discussion

ROS have been shown to have the dual effect of either suppressing or promoting tumor growth [15]. Here, we focused on the role of ROS derived from the NOX2 complex. This complex is known to be responsible for the respiratory burst in granulocytes, but is also expressed in phagocytes, antigen-presenting cells and MDSCs [24,26], cells which are of central importance for controlling host-anti tumor immunity. Furthermore several types of malignancies have been shown to overexpress NOX2, e.g. breast cancer and prostate cancer [17,19]. To study the role of NOX2-derived ROS we used a mouse strain in which a mutation in the NCF1 gene causes a loss of functional p46^phox^, a key organizational protein for the NOX2 complex [22]. This is the first study where this unique mutant has been used to analyze the effect on chemically induced sarcomas of NOX2 complex-derived ROS.

The MCA-induced tumor model is a prototype for studies of tumor immunology, in which the first tumor transplantation antigens were defined [3–5]. It was found that these antigens could be recognized by sensitize-isologous lymphoid cells[27], and further peptide specific responses by both CD4 as well as CD8 cells in the MCA model were identified [28]. This has further been confirmed to be T cell mediated tumor recognition using whole-exome sequencing and CTL epitope discovery [29]. Subsequently, this model has also been applied to studies of immune surveillance through the use of various immune deficient mouse strains (reviewed in [30]). MCA-induced tumors, when transplanted are able to oxidize thiols and increase the cystine/thiol ratio two fold above normal [31]. Treatment of these mice with antioxidants restored normal cystine/thiol ratios. In addition N-acetyl-cysteine was able to reduce tumor volume after one week of daily treatment [31]. We found the sarcomas initiated by MCA to develop in a similar manner in both NCF1*^/^* and NCF1*^/+^ mice. Since we could confirm that monocytic cells from NCF1*^/^* mice, in contrast to those from NCF1*^/+^ mice, were incapable of producing superoxide when stimulated, this argues against NOX2 derived ROS having a major role in MCA induced sarcoma development.

Chemical carcinogens induce extensive DNA damage, leading to the development of a high frequency of mutations that result in neo-antigens. As T cells reactive with neo-antigens have not been subjected to clonal elimination in the thymus, they are potentially able to generate a robust anti-tumor immune response which should be capable of controlling tumor development [29]. However, several mechanisms are antagonizing tumor rejection of MCA-induced tumors, including loss of tumor epitopes by clonal selection, leading to tumor escape variants [32,33]. Also, since the carcinogen induced development of sarcomas takes more than 160 days, this allows for long-term interaction of the tumor environment with the host immune system. Our previous studies have shown that mice bearing primary MCA induced tumors have diminished T cell and NK cell functions, impaired capacity to produce Th1 cytokines, and markedly reduced levels of the signal-transducing ζ chain in T cells and NK cells, similar to that described in cancer patients [34]. The underlying mechanism behind this immunosuppression in mice bearing MCA induced tumors involves the recruitment of suppressive immune cells such as MDSCs and T_regs_. In agreement with this and our results here (Fig 2), sarcomas have been shown to induce increases in circulating MDSC as shown in both humans and mice [24,35,36].

In contrast to the findings presented here, the growth of the transplantable mouse B16 melanoma and the Lewis lung carcinoma was, when tested in the same NCF1 mutant model, found to be significantly enhanced by NOX2 complex derived ROS [37]. Similarly to our model of MCA-induced sarcomas, the tumor growth in the spontaneously arising prostate carcinoma model (TRAMP) was not affected. Both these tumor models have in common a relatively long latency period before the manifestation of tumors, in contrast to the rapid development of the transplantable B16 melanoma and Lewis lung carcinoma lines. These findings were interpreted as ROS having a regulatory effect on the host inflammatory immune response to the transplanted tumors, in line with the earlier data on the effect of ROS on autoimmune inflammatory responses [26] in which a clear T cell response is apparent. In support of this interpretation, the lack of ROS-mediated protection against tumor growth was in the same study associated with increased production of immunity-associated cytokines. Sudden exposure of the mouse to transplanted and rapidly growing tumors are more likely to evoke a T cell response that could be regulated by ROS in comparison with models with slowly developing spontaneous or chemically induced tumors. Alternatively this might be due to the B16 and LLC tumor models inducing greater amounts of MDSCs [38] than the MCA induced sarcoma model. T_regs_ have previously been implicated in suppressing anti-tumor immunity, and in our MCA induced sarcoma model accumulation of high percentages of T_regs_ in both NCF1*^/^* as well as NCF1*^/+^ mice was found (Fig 2) while NOX2 was not key in regulation of tumorigenisis. In B16 and LLC mouse models variable percentages of T_regs_ were recruited [39,40], and in these models NOX2 did play a significant role [37]. This suggests that T_regs_ in these models may not suppress primarily through NOX2 mediated ROS.

MDSC are typically characterized as immature myeloid cells expressing CD11b+ Gr1+ in mice that effectuate their suppressive mechanisms through the production of ROS, arginase, indoleamine 2,3-dioxygenase, prostaglandins, and suppressive cytokines [9]. MCA-sarcomas induced a comparable increase of splenic MDSC in both NCF1*^/^* and NCF1*^/+^ mice. Additionally, the tumors contained an even higher number of MDSCs than did the spleens, but with no significant differences between the NCF1 mutant homozygous and heterozygous mice. MDSCs in mice have been reported to use the NOX2 complex to mediate their suppressive ROS function and high ROS were found to maintain the immature-suppressive phenotype of MDSC [24,41]. This appears to be in disagreement with our findings, where regardless of the NCF1 mutation status, MDSC´s increased both in the periphery and within the tumor. MyD88 has been shown to be required as well for the expansion of suppressive CD11b+Gr1+ cells [42], and MyD88^-/-^ mice developed MCA induced sarcomas with decreased frequency [7]. This further supports the role of MDSCs as a key facilitator of tumor immune escape. Seen in the context of our findings, this strongly suggests that while MDSCs seem to play an important role in tumor induced immunosuppression, their effect does not seem to be mediated through the NOX2 pathway in the MCA induced sarcoma model.

Similar to MDSCs, T_regs_ expressing CD25 and FoxP3 have been found to accumulate in tumors from both humans and mice and to exert their suppressive function through a multitude of factors including ROS, IL-10, and TGF-beta [8]. The MCA model promotes T_regs_ that are able to directly inhibit effector T cell function [43,44]. Depletion of CD25 positive cells in this model allows for rejection of tumors[45], indicating an important role for T_regs_ in suppressing anti-tumor immunity. While the MCA did elicit increased T_regs_ populations in the blood, spleen and tumor no difference was found in tumorigenesis due to non-functional NOX2 despite the large increase in this suppressive cell population; indicating that the key suppressive mechanism utilized by T_regs_ in this model is not ROS production. The T_reg_ mediated suppression may be limited to secretion of IL10 as mice with a knockout phenotype for this cytokine are less susceptible to MCA induced tumors [7].

ROS, as a mechanism of suppression, can inhibit T cell function [46,47]. We evaluated the ability of T cells to function and produce IFN-γ, IL-2, IL-4, and IL-17 upon short-term stimulation with PMA and found very few differences in functional T cell capacity between NCF1*^/^* and NCF1*^/+^ tumor bearing mice. Similarly, oxidative stress did not vary greatly between NCF1*^/^* and NCF1*^/+^ mice in spleen or tumor infiltrating T cells or MDSCs (Fig 5). This is in contrast to PMA activated monocytic cells that were able to produce large amounts of super-oxide upon stimulation in the NCF1*^/+^ mice (Fig 1A), suggesting that intratumoral MDSCs were not activated to produce ROS via NOX2 complex activation. There was a trend towards increased oxidative stress for T cells found in the tumor as compared to in the spleen, but this increase cannot be attributed to functional NOX2 (Fig 5) and may be due to metabolic induced oxidative stress as has been hinted at by transplant studies using MCA-induced sarcomas [31]. The main source of oxidative stress in another slowly growing malignancy, namely CLL, has been shown to be mitochondrial in origin. The treatment of CLL with antioxidants and molecules inhibiting oxidative phosphorylation lead to specific killing of the malignancy [48].

Previously it has been demonstrated that MCA generated sarcomas undergo immune editing when developing in WT mice, but not when developing in RAG^-/-^ mice, leading to rejection of RAG^-/-^ initiated tumors but not WT initiated ones [49]. We therefore asked if the lack of NOX2 produced ROS would affect the capacity of MCA induced tumors to grow in WT mice by transplanting tumors generated in both NCF1*^/^* and NCF1*^/+^ into wild type C57Bl/6 mice. Although a slight trend towards increased tumor take of sarcomas transplanted from NCF1*^/^* mutant mice was observed, this difference was not significant and did not support a role for NOX2 produced ROS in immune editing of MCA induced sarcomas.

Oxidative stress in tumors drive many of the hallmarks of cancer; including proliferation, invasion and resistance to therapy [10]. To further address the role of NOX2 in tumorigenesis we established and characterized 15 tumor cell lines. We found that tumor lines generated in NCF1*^/^* and NCF1*^/+^ mice produced similar amounts of free radicals. Their growth and invasive phenotypes were uniformly found to be independent of NCF1 mutation status. While these tumors were quite resistant to clinically relevant radiation doses, displaying some growth inhibition, and alternatively quite sensitive the platinum based chemotherapies; this was not influenced by the NCF1 genotype.

In autoimmune diseases, a regulatory role of oxidative stress and its suppressive function on immune activation has been well established although the underlying mechanisms are still to be delineated [50]. The complexities involved in tumor and immune cell interactions hint at a similar function in this context, but as of yet the findings indicate that there is much to uncover about NAPH oxidase induced oxidative stress. While oxidative stress has long been known to be an essential key in tumorigenesis, its rout of generation may differ per tumor type. Identifying in which tumors NOX2 mediated oxidative stress is essential will allow for the appropriate application related therapies.

## Supporting Information

S1 Fig Gating of monocytic cells based on size and granulation after acquisition on LSRII flowcytometer. (Left panel) Spleenocytes harvested from non-tumorbearing NCF1*^/^* and NCF1*^/+^ mice were labeled with DHR123 prior to stimulation with PMA followed by acquisition.
(TIFF)

S2 Fig T_regs_ increase in the spleen and blood in tumor bearing mice. (A) T_regs_ were gated as follows: by gating singlet cells, followed by live cells and CD4+ CD3+ cells, prior to gating on FoxP3. (Bi) Percent T_reg_ cells in the blood, spleen and tumor of NCF1*^/^* MCA tumor bearing mice; * p < 0.05. (Bii) Percent T_reg_ cells in the blood, spleen and tumor of NCF1*^/+^ MCA tumor bearing mice; * p < 0.05, ** p < 0.01, *** p < 0.001.
(TIFF)

S3 Fig T cell activation and memory phenotypes. (A) Activation of T cells through acquisition of CD69 labeled blood, spleenocytes and tumors on LSRII; * p < 0.05, ** p < 0.01, *** p < 0.001. (B) Memory phenotype of T cells through acquisition of CD44 and CD62L labeled blood, spleenocytes and tumors on LSRII; * p < 0.05, ** p < 0.01, *** p < 0.001.
(TIFF)

S4 Fig Ratio of Th1, as determined by IFN-γ positive CD4 T cells, to Th2 cells, as determined by IL-4 positive CD4 T cells in the blood, spleen and tumor of NCF1*^/^* and NCF1*^/+^ tumor bearing mice; * p < 0.05. (TIFF)
