# Supplementary figures and images for: Methylcholanthrene-Induced Sarcomas Develop Independently from NOX2-Derived ROS
Source: PLoS One. 2015 Jun 15;10(6):e0129786. doi: 10.1371/journal.pone.0129786 (PMC4468117; doi:10.1371/journal.pone.0129786)

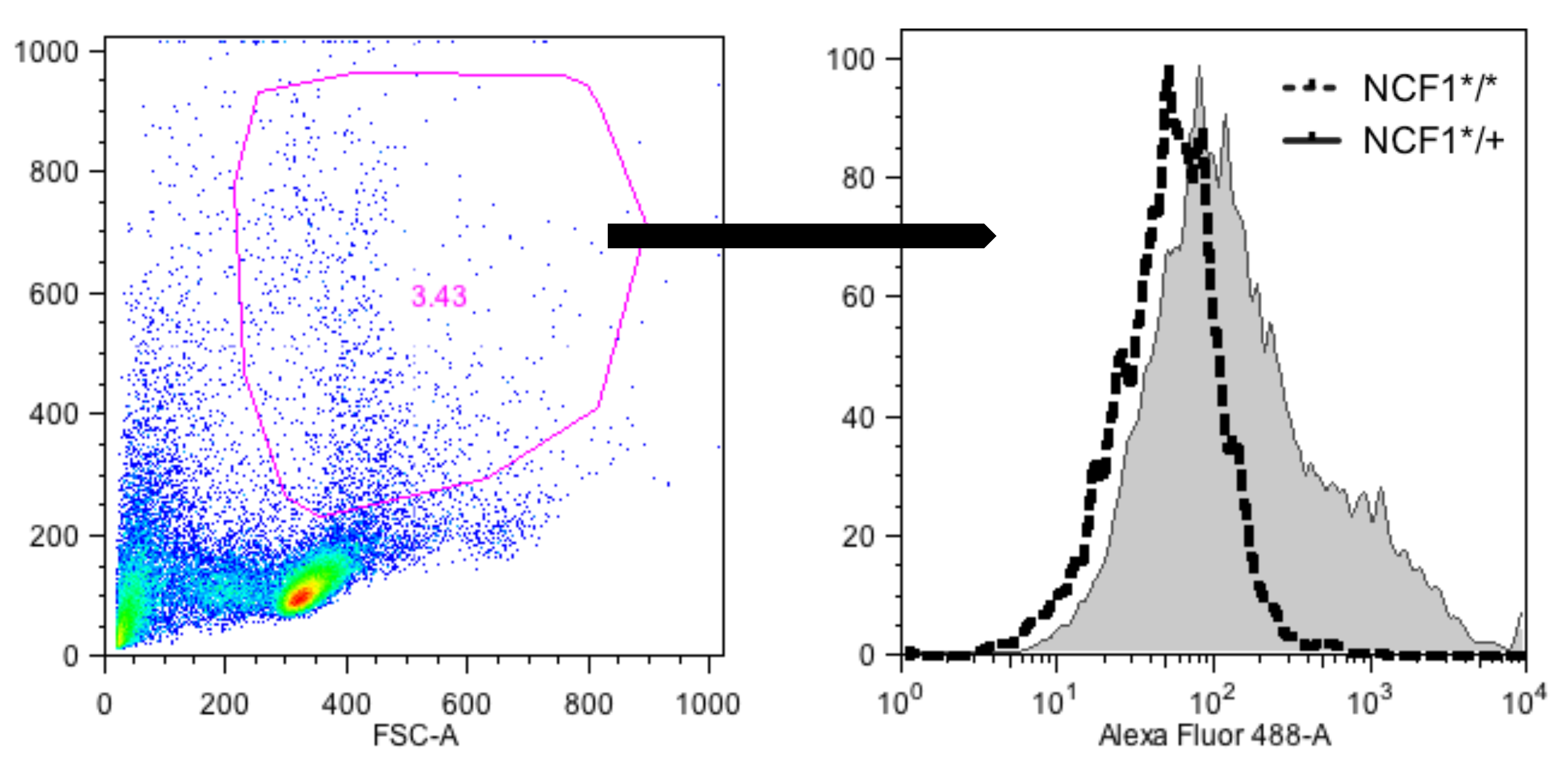

Supplement: S1 Fig — (Left panel) Spleenocytes harvested from non-tumorbearing NCF1*/* and NCF1*/+ mice were labeled with DHR123 prior to stimulation with PMA followed by acquisition. (TIFF) [file pone.0129786.s001.tiff]

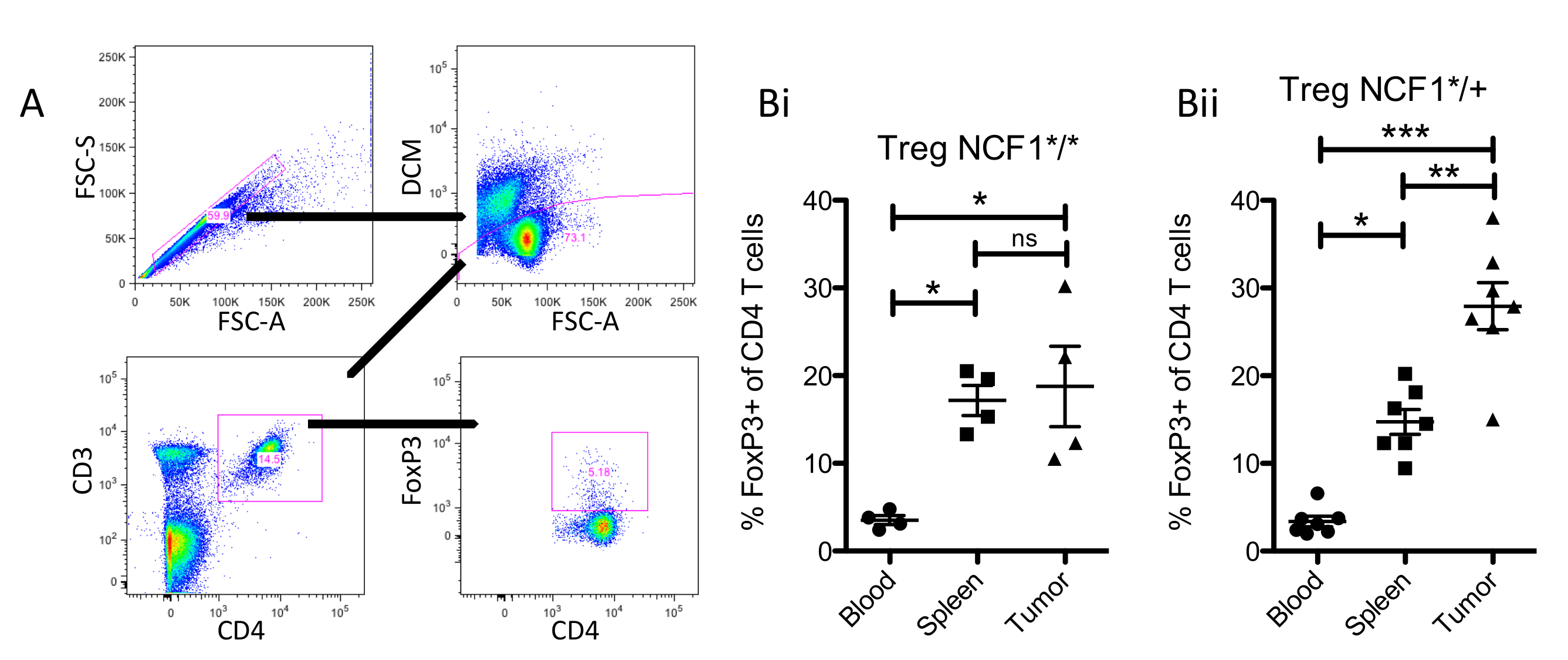

Supplement: S2 Fig — (A) Tregs were gated as follows: by gating singlet cells, followed by live cells and CD4+ CD3+ cells, prior to gating on FoxP3. (Bi) Percent Treg cells in the blood, spleen and tumor of NCF1*/* MCA tumor bearing mice; * p < 0.05. (Bii) Percent Treg cells in the blood, spleen and tumor of NCF1*/+ MCA tumor bearing mice; * p < 0.05, ** p < 0.01, *** p < 0.001. (TIFF) [file pone.0129786.s002.tiff]

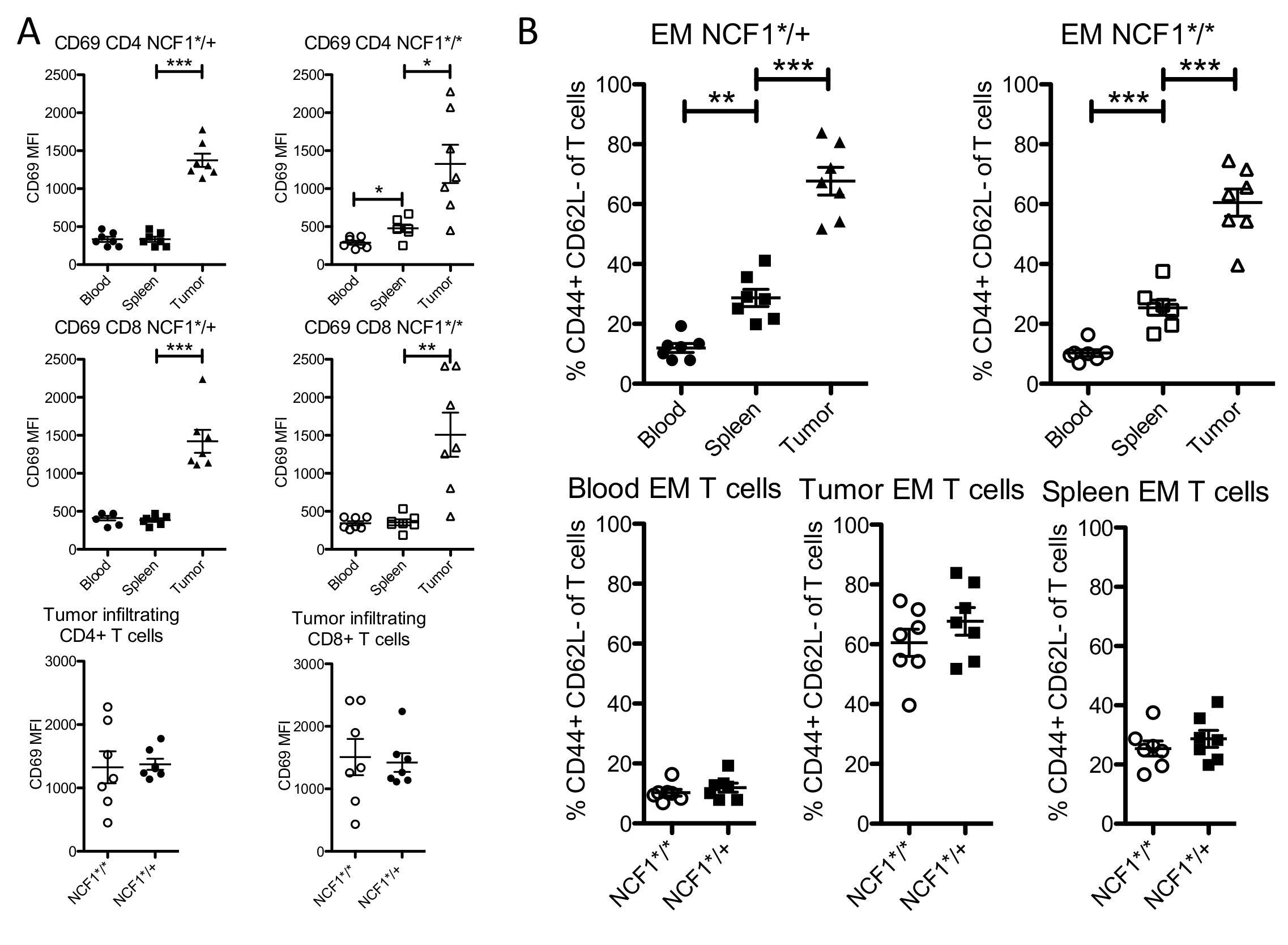

Supplement: S3 Fig — (A) Activation of T cells through acquisition of CD69 labeled blood, spleenocytes and tumors on LSRII; * p < 0.05, ** p < 0.01, *** p < 0.001. (B) Memory phenotype of T cells through acquisition of CD44 and CD62L labeled blood, spleenocytes and tumors on LSRII; * p < 0.05, ** p < 0.01, *** p < 0.001. (TIFF) [file pone.0129786.s003.tiff]

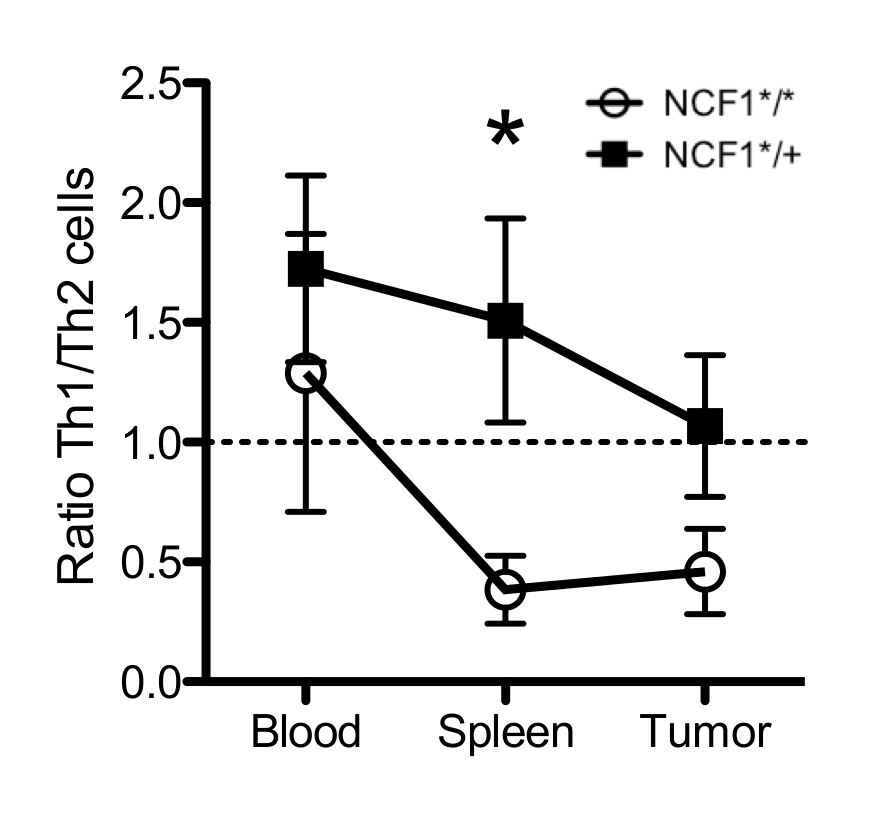

Supplement: S4 Fig — (TIFF) [file pone.0129786.s004.tiff]
